# Phenotypic and molecular characterization of extended spectrum- and metallo- beta lactamase producing *Pseudomonas aeruginosa* clinical isolates from Egypt

**DOI:** 10.1007/s15010-024-02297-8

**Published:** 2024-06-02

**Authors:** Eva A. Edward, Marwa R. El Shehawy, Alaa Abouelfetouh, Elsayed Aboulmagd

**Affiliations:** 1 Department of Microbiology and Immunology, Faculty of Pharmacy, Alexandria University, El-Khartoom Square, Azarita, Alexandria, Egypt; 2 Department of Microbiology and Immunology, Faculty of Pharmacy, Alamein International University, Alamein, Egypt; 3 College of Pharmacy, Arab Academy for Science, Technology and Maritime, Alamein Branch, Alamein, Egypt

**Keywords:** *bla*_OXA-10_, *bla*_VIM_, Genotype combinations, Curing, Transformation

## Abstract

**Background:**

Antimicrobial resistance among *Pseudomonas aeruginosa* (*P. aeruginosa*), a leading cause of nosocomial infections worldwide, is escalating. This study investigated the prevalence of extended-spectrum β-lactamases (ESBLs) and metallo-β-lactamases (MBLs) among 104 *P. aeruginosa* clinical isolates from Alexandria Main University Hospital, Alexandria, Egypt.

**Methods:**

Antimicrobial susceptibility testing was performed using agar dilution technique, or broth microdilution method in case of colistin. ESBL and MBL prevalence was assessed phenotypically and genotypically using polymerase chain reaction (PCR). The role of plasmids in mediating resistance to extended-spectrum β-lactams was studied via transformation technique using plasmids isolated from ceftazidime-resistant isolates.

**Results:**

Antimicrobial susceptibility testing revealed alarming resistance rates to carbapenems, cephalosporins, and fluoroquinolones. Using PCR as the gold standard, phenotypic methods underestimated ESBL production while overestimating MBL production. Eighty-five isolates (81.7%) possessed only ESBL encoding genes, among which 69 isolates harbored a single ESBL gene [*bla*_OXA-10_ (n = 67) and *bla*_PER_ (n = 2)]. Four ESBL-genotype combinations were detected: *bla*_PER_ + *bla*_OXA-10_ (n = 8), *bla*_VEB-1_ + *bla*_OXA-10_ (n = 6), *bla*_PSE_ + *bla*_OXA-10_ (n = 1), and *bla*_PER_ + *bla*_VEB-1_ + *bla*_OXA-10_ (n = 1). Three isolates (2.9%) possessed only the MBL encoding gene *bla*_VIM_. Three ESBL + MBL- genotype combinations: *bla*_OXA-10_ + *bla*_AIM_, *bla*_OXA-10_ + *bla*_VIM_, and *bla*_PER_ + *bla*_OXA-10_ + *bla*_AIM_ were detected in 2, 1 and 1 isolate(s), respectively. Five plasmid preparations harboring *bla*_VEB-1_ and *bla*_OXA-10_ were successfully transformed into chemically competent *Escherichia coli *DH5α with transformation efficiencies ranging between 6.8 × 10 3 and 3.7 × 10 4    CFU/μg DNA plasmid. Selected tested transformants were ceftazidime-resistant and harbored plasmids carrying *bla*_OXA-10_.

**Conclusions:**

The study highlights the importance of the expeditious characterization of ESBLs and MBLs using genotypic methods among *P. aeruginosa* clinical isolates to hinder the development and dissemination of multidrug resistant strains.

**Supplementary Information:**

The online version contains supplementary material available at 10.1007/s15010-024-02297-8.

## Background

*Pseudomonas aeruginosa* (*P. aeruginosa*), a clinically significant Gram-negative bacterium belonging to the ESKAPE organisms, has been recognized as a leading cause of serious nosocomial infections worldwide [1]. *P. aeruginosa* can cause both acute and chronic infections such as ventilator-associated pneumonia, abscesses, skin and soft tissue infections, septic arthritis, bacteremia, meningitis, ulcerative keratitis, and conjunctival erythema. *P. aeruginosa* infections are more serious in immunocompromised individuals, such as acquired immunodeficiency syndrome patients, cystic fibrosis patients, and patients receiving chemotherapies [2–4].

Carbapenems, aminoglycosides, fluoroquinolones, and cephalosporins have been used for treatment of *P. aeruginosa* infections. However, resistance of *P. aeruginosa* to various antimicrobial agents is increasing in many countries. Ceftazidime is the most routinely prescribed cephalosporin for combating *P. aeruginosa* infections owing to its distinctive anti-pseudomonal activity, yet, ceftazidime resistance is escalating [5].

Antimicrobial resistance has rendered *P. aeruginosa* infections life-threatening and hard to treat [6], because of the limited therapeutic options remaining. This results in worse outcomes and higher morbidity and mortality, particularly in those with severe *P. aeruginosa* infections [7]. Previous treatment with antibiotics showing high antipseudomonal activity as well as prolonged duration of antibiotic treatment are among the major risk factors contributing to the emergence of resistant *P. aeruginosa* strains [8].

*P. aeruginosa* can develop resistance to different antibiotics though intrinsic or acquired resistance mechanisms [9]. Intrinsic resistance occurs due to expression of efflux pump systems, decreased outer membrane permeability, and production of antibiotic-inactivating enzymes [10]. In addition, *P. aeruginosa* acquires resistance to antimicrobials through mutational changes and acquisition of resistance genes via horizontal gene transfer. Transfer of genes encoding extended-spectrum β-lactamases (ESBLs) and metallo-β-lactamases (MBLs), through conjugation, transduction, and transformation, is the main contributor to the dissemination of antimicrobial resistance among *P. aeruginosa* strains [9].

Various ESBLs, belonging to molecular classes A and D, have been identified in *P. aeruginosa* [11]. Class A ESBLs, e.g. TEM, VEB, PER, SHV, GES, and BEL [12], mediate resistance to penicillins as well as narrow spectrum and third generation cephalosporins, but not the carbapenems [11]. The activity of Class A ESBLs may be inhibited by clavulanic acid as well as tazobactam [13]. Class D ESBLs, known as OXA-type ESBLs, resist inactivation by β-lactamase inhibitors except for OXA-18 and OXA-45 [13]. *P. aeruginosa* also produces different MBLs, including AIM, FIM, GIM, IMP, NDM, SPM, and VIM. They belong to class B β-lactamases, and can break down most β-lactam antibiotics, including carbapenems [5, 14, 15].

An alarming widespread detection of ESBL genes among Gram-negative pathogens in Egypt and surrounding countries; such as Saudi Arabia and Sudan; has been recorded [16]. Also, a recent meta-analysis in Egypt, published in 2022, has revealed an elevated prevalence of MBL-producing *P. aeruginosa* reaching about 33.7% [17]. Furthermore, the co-expression of ESBLs and MBLs has been reported among clinical isolates, emphasizing the necessity of their rapid detection to establish a suitable policy focusing on restricting empirical antibiotics’ prescription [5, 6].

This study aimed to investigate the prevalence of ESBLs and MBLs, both phenotypically and genotypically, among *P. aeruginosa* clinical isolates collected from Alexandria Main University Hospital (AMUH), the largest tertiary hospital in Alexandria, Egypt. In addition, the role of plasmids in mediating resistance to extended-spectrum β-lactams was studied via curing and transformation experiments using plasmids isolated from selected ceftazidime-resistant *P. aeruginosa* clinical isolates, a nationally and globally reported challenging and disseminating pathogen.

## Methods

### Bacterial isolates

In the present study, 104 *P. aeruginosa* clinical isolates previously collected from different clinical specimens (pus n = 56, bronchial lavage n = 25, urine n = 13, sputum n = 8, and blood cultures n = 2) from the medical microbiology lab at  AMUH, Alexandria, Egypt, between September 2017 and November 2017 and from June 2018 to October 2018 [18] were included. The collected isolates were previously identified [18] through Gram staining, growth on cetrimide agar plates (Himedia, India), triple sugar iron (TSI) test, growth at 42 °C, and the oxidase test (Himedia, India) [19].

### Antimicrobial susceptibility testing

The minimum inhibitory concentrations (MICs) of piperacillin-tazobactam, ceftazidime, cefepime, imipenem, meropenem, gentamicin, ciprofloxacin, levofloxacin, and moxifloxacin against the tested isolates were determined using the agar dilution technique, whereas MIC of colistin was determined using the broth microdilution technique [20] and the results were interpreted according to CLSI, 2018 [21]. The antibiotic powders/solutions of pharmaceutical grade were purchased from the Egyptian market as follows: piperacillin-tazobactam (Tazocin^®^ 4.5 g, Pfizer pharmaceutical Co.); ceftazidime (Inzad^®^1g, Rameda & Marcyrl Co.); cefepime (Cefepime^®^ 1g, Pharco B International); imipenem (Tienam^®^500 mg, Merck Sharp & Dohme B.V.); meropenem (Meronem^®^ 500 mg, Astrazeneca, UK); ciprofloxacin (Ciprofloxacin^®^ 200 mg/100 ml infusion, AMRYIA. IND); levofloxacin (Tavanic^®^ 500 mg/100 ml infusion, Sanofi Aventis); and moxifloxacin (Vigamox^®^ 5 mg/ml eye drops, Alcon, Geneva). Colistin was obtained as colistin sulfate powder (Amoun Pharmaceutical Co., Egypt) and gentamicin was obtained as gentamicin sulfate powder (Sigma -Alderich, Germany).

The resistance score (R score), designated as the number of antibiotics to which the isolate showed resistance, was calculated for each tested strain. Each resistant call was given a score of 1, while an intermediate resistance towards the tested antibiotic was given a score of 0.5 [22].

### Phenotypic detection of β-lactamases

#### Detection of ESBLs

##### Double disc synergy test (DDST)

A disc of amoxicillin-clavulanate (20/10 µg) was placed 30 mm apart (center to center) from a disc containing ceftazidime (30 µg) or cefepime (30 µg) [Himedia, India] on a Müller-Hinton agar (LAB M, UK) plate swabbed with the overnight culture of each tested isolate and the plate was incubated at 37 °C for 24 h. ESBL production was considered positive if the inhibition zone around cephalosporin discs (ceftazidime or cefepime) was extended on the side towards the amoxicillin-clavulanate disc [23].

##### Phenotypic confirmatory disc diffusion test (PCDDT)

Discs containing ceftazidime (30 µg) alone and in combination with clavulanic acid (10 µg) [Himedia, India] were placed 30 mm apart (center to center) on Müller-Hinton agar plate swabbed with the overnight culture of the tested isolate. The plates were incubated at 37 °C for 24 h. An increase of 5 mm in the inhibition zone diameter around the ceftazidime-clavulanate disc relative to the ceftazidime disc was indicative of ESBL production [21, 24].

#### Detection of MBLs

##### Imipenem-EDTA combined disc test (IPM-EDTA CDT)

Overnight cultures of the tested isolates were swabbed on Müller-Hinton agar plates. Discs of imipenem (10 μg) and imipenem-EDTA (10/750 μg) [Himedia, India] were placed on the plates 20 mm apart (center to center). The inhibition zone diameters of the imipenem and imipenem-EDTA discs were compared after 24 h of incubation at 37 °C. An increased inhibition zone of ≥ 7 mm with the imipenem-EDTA disc compared to the imipenem disc alone was considered a positive indication for MBL production [25].

### Molecular detection of β-lactamases

#### Detection of genes encoding selected β-lactamases

For the preparation of DNA templates from the tested clinical isolates, four colonies of each isolate were suspended in 200 μl of sterile water for injection in an Eppendorf tube. The suspension was heated at 95 °C for 30 min and then frozen at – 20 °C for 30 min. After thawing, the tube was centrifuged at 14,000 rpm for 10 min, then preserved as aliquots at -20 °C for future use [26, 27]. To isolate DNA from transformed cells, a single colony was suspended in 5 μl sterile water for injection and heated at 95 °C for 10 min to lyse the cells [28]. The presence of selected β-lactamase genes: class A ESBL genes (*bla*_PER_, *bla*_PSE_ and *bla*_VEB-1_), class B MBL genes (*bla*_IMP_, *bla*_VIM_, *bla*_AIM_ and *bla*_NDM_), and class D ESBL genes (*bla*_OXA-10_) was tested in the extracted DNA by the polymerase chain reaction (PCR) using previously published primers [29–32] purchased from Thermo-Scientific (USA). The used primers’ sequences and the applied thermocycling conditions are listed in Additional file 1: Tables S1 and S2, respectively. The amplified PCR products were resolved using 1% agarose gel electrophoresis in Tris Acetate EDTA (TAE) buffer (40 mM Tris, 1 mM EDTA, and 20 mM acetic acid, pH 8.5), and the bands were visualized at 254 nm, using a UV trans-illuminator (Entela, USA). A 100 bps DNA ladder was run alongside the PCR products to detect band size (GeneDirex, Taiwan).

### Plasmid isolation and characterization

Plasmid isolation from the ceftazidime-resistant *P. aeruginosa* clinical isolates harboring both *bla*_OXA-10_ and *bla*_VEB-1_ (P23, P78, P100, P101, P108, P121, and P123) was performed using the plasmid isolation kit "QIAprep® Plasmid Miniprep Kit" (Qiagen, Germany) according to the manufacturer’s instructions. The concentrations of the obtained plasmids were measured using Genova nano micro-volume spectrophotometer (Jeneway, UK). The plasmid extract was used as a DNA template for PCR detection of the plasmid mediated genes, *bla*_VEB-1_ and *bla*_OXA-10_, as previously described. The extracted plasmids were resolved on 0.8% agarose gel electrophoresis in the presence of 1 Kbp DNA ladder to describe the plasmid profile (GeneDirex, Taiwan).

### Transformation of chemically competent *Escherichia coli *cells with plasmids harboring *bla*_VEB-1_ and *bla*_OXA-10_

The extracted plasmid preparations, carrying both *bla*_VEB-1_ and *bla*_OXA-10_, were transformed into chemically competent *E. coli* cells using heat shock technique [33]. *E. coli* DH5α chemically competent cells were prepared as previously described [33]. Briefly, frozen Eppendorf tubes containing 50 μl of chemically competent *E. coli* DH5α cells were thawed on ice for 15 min. Five microliters of each plasmid preparation were added to a tube containing the thawed competent cells, mixed thoroughly, and incubated on ice for 15 min. The cells were incubated for 90 s in a pre-adjusted heat block at 42 °C, and then transferred back to ice for 5 min. Nine hundred microliters of Luria–Bertani (LB) broth were added to each Eppendorf tube and the cells were incubated at 37 °C with shaking for 1 h at 160 rpm to recover the bacterial cells. From each culture, 50 μl were aseptically plated onto sterile LB agar plate containing 0.5 × MIC of ceftazidime for each selected isolate. After incubation at 37 °C for 24 h, the plates were checked for any transformants. For further confirmation, the susceptibility of   selected resultant transformants as well as *E. coli* DH5α to ceftazidime was detected by the disc diffusion method [21], using ceftazidime (CAZ, 30) disc (Himedia, India), as well as MIC determination by agar dilution method [20]. Selected transformants were subjected to plasmid isolation, then the plasmid extracts were used as templates for PCR amplification of *bla*_VEB-1_ and *bla*_OXA-10_ genes as previously described.

Transformation efficiency (no. of transformants per μg of plasmid DNA) was calculated as follows [34]:

$${\text{Transformation \, efficiency}} = \frac{{{\text{Total \, number \, of \, transformants \, growing \, on \, the \, agar \, plate \, containing}}\, 0.5 \times {\text{MIC \, of \, ceftazidime}} }}{{{\text{Amount \, of \, plasmid DNA}} \,({\text{in}} \, \upmu {\text{g}}) }}$$

### Curing of resistance plasmids from selected *P. aeruginosa* clinical isolates

Plasmid curing experiment was performed for the seven selected *P. aeruginosa* isolates using ethidium bromide (EtBr) [100 μg/ml and 300 μg/ml] and sodium dodecyl sulfate (SDS) [5% and 10%] as curing agents. These agents were sterilized by filtration using cellulose acetate syringe filters (Pore size: 0.45 µm, Diameter: 13 mm) [Filter-bio, China]. The selected isolates were grown in LB broth at 37 ºC for 24 h. One hundred microliters of each culture were added to 2 ml LB broth containing the different concentrations of the curing agents and incubated at 37 ºC for 24 h with constant shaking at 180 rpm. The cultures were ten-fold serially diluted with 0.9% sterile saline and then an aliquot of 100 μl of each dilution was plated onto a sterile nutrient agar plate to obtain separate colonies. The plates were incubated at 37 ºC for 24 h. About 150–200 representative colonies were picked up onto control LB agar plate as well as LB agar plates containing sub-inhibitory concentrations (0.5 × MIC) of the marker antibiotics (ceftazidime, imipenem, gentamicin, and levofloxacin) for each selected isolate. The plates were incubated at 37 ºC for 24 h, and then examined for the growth of the cured colonies [35, 36]. The cells that failed to grow on antibiotic-containing plates while still being able to grow on the antibiotic-free plate were considered cured.

The curing rate was calculated as follows [37]:

$${\text{Curing \, rate }} = \frac{{{\text{No}}{\text{. \, of \, cured \, colonies }}}}{{{\text{Total \, No}}{\text{. \, of \, tested \, colonies}}}} \times 100$$

The cured colonies were picked from the control plates and checked for their susceptibility towards the tested antimicrobial agents by the disc diffusion method [21] using the following antibiotic discs (Himedia, India): ceftazidime (CAZ, 30), imipenem (IPM, 10), gentamicin (GEN, 10), and levofloxacin (LE, 5).

### Sequencing of the *bla*_VEB-1_ gene

*bla*_VEB-1_ was amplified from the seven selected *P. aeruginosa* clinical isolates using PCR. The PCR fragments were purified, and then sequenced using the same forward and reverse primers used for the PCR protocol (Additional file 1: Table S1). PCR fragment purification and nucleotide sequencing was conducted at GATC Biotech DNA sequence company (Germany, https://www.genomeweb.com/companies/gatc-biotech) using Sanger sequencing technology. The sequences were aligned against the sequence of *bla*_VEB-1a_ (GenBank accession no. HM370393.1) using the Basic Local Alignment Search Tool (BLAST) to confirm the sequence identity.

### Statistical analysis

Data were analyzed using the Chi-square test analysis using SPSS version 25 for windows (SPSS Inc., Chicago, IL, USA). To study the association between susceptibility to different pairs of antimicrobial agents, data were analyzed using IBM SPSS software package version 20.0. (IBM Corp., Armonk, NY, USA). Spearman’s correlation was applied to construct the correlation matrix. After calculating the *p*-values, the significance of the obtained results was judged at the 5% level.

## Results

### Antimicrobial susceptibility testing

A total of 104* P. aeruginosa* clinical isolates, collected from AMUH, were included in this study. The antimicrobial susceptibility testing revealed that the collected isolates displayed elevated resistance to imipenem (86.5%), the fluoroquinolones: ciprofloxacin, levofloxacin, and moxifloxacin (85.6, 84.6, and 83.7%, respectively), cefepime and gentamicin (82.7% each), followed by meropenem (76%) and ceftazidime (67.3%). On the other hand, colistin resistance was minimal where 94.2% of the tested isolates were colistin-susceptible. For piperacillin-tazobactam, 27.9% of the isolates were susceptible and the remaining isolates showed intermediate (33.7%) to complete resistance (38.5%) **(**Table 1). Among the tested isolates, 3.8% (n = 4) recorded the highest R score of 10 while 28.8% of the isolates (n = 30) exhibited R score of 9. On the other hand, 4 isolates (3.8%) were susceptible to all of the tested antibiotics recording R score of 0. The antibiotic resistance profile and the R score of each of the tested isolates are illustrated in Additional file 1: Table S3.

**Table 1:** Antimicrobial susceptibility of the tested *P. aeruginosa* clinical isolates

Antimicrobial agent	MIC^a^ (µg/ml)			Susceptibility^d^ (%)		
	Range	MIC_50_^b^	MIC_90_^c^	%S	%I	%R
Piperacillin-tazobactam	4—> 1024	64	256	27.9	33.7	38.5
Ceftazidime	< 0.5— >256	64	>256	25	7.7	67.3
Cefepime	< 0.5— >1024	64	512	15.4	1.9	82.7
Imipenem	< 0.5— >512	32	>512	10.6	2.9	86.5
Meropenem	< 0.5—> 256	64	>256	18.3	5.8	76
Gentamicin	1— >1024	1024	>1024	16.3	1	82.7
Ciprofloxacin	<0.5— >128	16	64	14.4	0	85.6
Levofloxacin	<0.5— >128	64	128	15.4	0	84.6
Moxifloxacin	<0.5— >128	64	> 128	16.3	0	83.7
Colistin	<0.5—256	<0.5	2	94.2	0	5.8

^a^MIC: minimum inhibitory concentration

^b^MIC_50_: MIC of the antimicrobial agent required to inhibit the growth of 50% of the tested clinical isolates

^c^MIC_90_: MIC of the antimicrobial agent required to inhibit the growth of 90% of the tested clinical isolates

^d^S: sensitive, I: intermediate, and R: resistant

Among β-lactam antibiotics, piperacillin- tazobactam showed the highest MIC range (4 to  >1024 µg/ml). The three tested fluoroquinolones: ciprofloxacin, levofloxacin, and moxifloxacin had the same MIC range (< 0.5 to  >128 µg/ml). The MIC values of gentamicin and colistin ranged from 1 to  >1024 µg/ml, and from  <0.5 to 256 µg/ml, respectively (Table 1).

Gentamicin showed the highest MIC_50_ and MIC_90_ (1024 and > 1024 µg/ml, respectively). On the contrary, colistin exhibited the lowest MIC_50_ and MIC_90_ (< 0.5 and 2 µg/ml, respectively). Regarding β-lactam antibiotics, piperacillin-tazobactam, ceftazidime, cefepime, and meropenem possessed equal MIC_50_ values (64 µg/ml). The highest MIC_90_ was recorded for imipenem (> 512 µg/ml), while the lowest was observed in case of piperacillin-tazobactam (256 µg/ml). Amongst the tested fluoroquinolones, ciprofloxacin possessed the lowest MIC_50_ and MIC_90_ (16 and 64 µg/ml, respectively). Both levofloxacin and moxifloxacin had the same MIC_50_ (64 µg/ml) **(**Table 1**).**

**Fig. 1:**
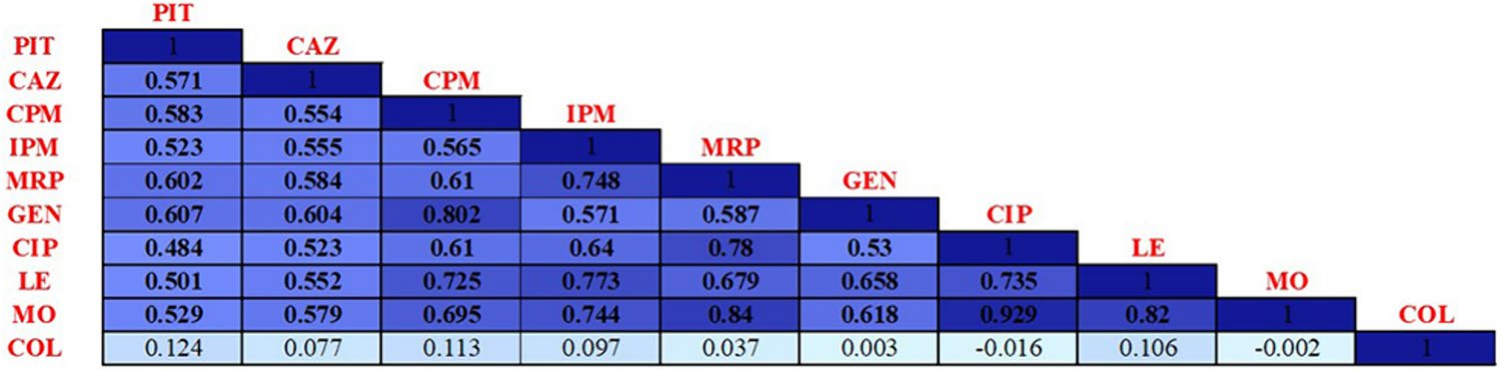
Correlation matrix showing Spearman’s correlation coefficients (r_s_) for each pair of antibiotics calculated according to the resistance patterns of 104 tested *P. aeruginosa* clinical isolates. The boldness of the blue color refers to the strength of the relationship between antibiotics, with stronger correlations having bolder colors. Numbers within boxes indicate correlation coefficient (r_s_) values. Spearman’s correlation coefficients (r_s_) written in bold indicate statistically significant levels of correlation at *p*-value ≤ 0.001. PIT piperacillin-tazobactam, CAZ ceftazidime, CPM cefepime, IPM imipenem, MRP meropenem, GEN gentamicin, CIP ciprofloxacin, LE levofloxacin, MO moxifloxacin, COL colistin

Based on the susceptibility data of the collected *P. aeruginosa* clinical isolates, illustrated in Additional file 1: Table S3, a correlation matrix relating the ten tested antimicrobial agents included in this study was established (Fig. 1). The matrix indicated that the highest correlations were observed in the resistance pattern of moxifloxacin and each of ciprofloxacin, meropenem, and levofloxacin (Spearman’s correlation coefficient r_s_ = 0.929, 0.84 and 0.82, respectively) as well as in case of the resistance pattern of gentamicin and cefepime (r_s_ = 0.802). All these relations were highly significant (*p*-value < 0.001). Six pairs of antibiotics showed highly significant correlations (*p*-value < 0.001) with r_s_ ranging between 0.78 and 0.725. These correlations were between the resistance patterns of ciprofloxacin and meropenem; imipenem and levofloxacin; imipenem and meropenem; imipenem and moxifloxacin; ciprofloxacin and levofloxacin; and cefepime and levofloxacin. All remaining Spearman’s correlation coefficients were also greater than 0.5 (r_s_ ranging between 0.695 and 0.501) showing significance at 0.001 level except for the correlation between the resistance pattern of ciprofloxacin and piperacillin-tazobactam (r_s_ = 0.484; *p*-value < 0.001) as well as the correlations between the resistance pattern of colistin and all other tested antibiotics (non-significant correlations with r_s_ ≤ 0.124).

The highest percentage of antimicrobial resistance among pus isolates was towards gentamicin, ciprofloxacin, and levofloxacin (85.7% each). The least effective antibiotics against bronchial lavage and urine isolates were imipenem (96 and 92.3% resistance, respectively), followed by the tested fluoroquinolones (88 and 84.6% resistance for each, respectively). Cefepime showed a similar activity (88% resistance) as the fluoroquinolones against bronchial lavage isolates. Both fluoroquinolones, ciprofloxacin, and moxifloxacin, exhibited the highest percentage of antimicrobial resistance (75% for each) among sputum isolates. Both blood isolates were resistant to cefepime, gentamicin as well as the tested carbapenems and fluoroquinolones. On the contrary, colistin was the most effective antibiotic against isolates from all clinical sources (Additional file 1: Table S3).

### Phenotypic detection of β-lactamases

None of the tested isolates showed positive results when phenotypically tested for ESBL production using DDST or PCDDT. The production of MBL was phenotypically detected among 87.5% of the isolates using the IPM-EDTA CDT. All imipenem-resistant isolates were found to be MBL producers. 

### Molecular detection of ESBL- and MBL-encoding genes

The three tested genes belonging to class A ESBLs; *bla*_PER_, *bla*_VEB-1_ and *bla*_PSE_ were detected among 11.5, 6.7 and 1% of the tested isolates, respectively. Among class B, only *bla*_VIM_ and *bla*_AIM_ MBLs were detected with prevalence rates of 3.8 and 2.9%, respectively. The tested member of class D β-lactamases, *bla*_OXA-10_, showed the highest occurrence rate (83.7%) among the tested isolates. Only two isolates: P23 and P93 harbored three genes each: P23 harbored *bla*_PER_, *bla*_VEB_, and *bla*_OXA-10_; and P93 harbored *bla*_PER_, *bla*_AIM_, and *bla*_OXA-10_. On the other hand, 12 isolates possessed none of the tested genes (Additional file 1: Table S3).

Table 2 illustrates the frequency of ESBL and MBL genes among *P. aeruginosa* clinical isolates. Eighty-five isolates (81.7%) possessed only ESBL encoding genes, among which 69 isolates harbored a single ESBL gene while 16 isolates harbored a combination of two or more ESBL genes. Regarding the single ESBL genotype, *bla*_OXA-10_ and *bla*_PER_ were detected among 64.4 and 1.9% of the isolates, respectively. The most common ESBL-genotype combinations were *bla*_PER_ + *bla*_OXA-10_ followed by *bla*_VEB-1_ + *bla*_OXA-10_ (recorded among 7.7 and 5.8% of the isolates, respectively). Each of *bla*_PSE_ + *bla*_OXA-10_ and *bla*_PER_ + *bla*_VEB-1_ + *bla*_OXA-10_ ESBL-genotype combinations was observed in one isolate (1%). Three isolates (2.9%) possessed only an MBL encoding gene ( *bla*_VIM_). Three ESBL + MBL- genotype combinations: *bla*_OXA-10_ + *bla*_AIM_, *bla*_OXA-10_ + *bla*_VIM_, and *bla*_PER_ + *bla*_OXA-10_ + *bla*_AIM_ were detected in 2, 1 and 1 isolate(s), respectively.* bla*_VEB-1_, *bla*_PSE_ and *bla*_AIM_ were always detected in the presence of other beta-lactamases.

**Table 2:** Frequency of ESBL and MBL encoding genes among *P. aeruginosa* clinical isolates

Type of resistant determinant (no. of isolates)	Explored genes	No. of isolates	Distribution (%)
ESBL^a^ encoding genes only (85)			
Single ESBL encoding gene (69)	*bla*_PER_	2	1.9
	*bla*_OXA-10_	67	64.4
Two or more ESBL encoding genes (16)	*bla*_PER_ + *bla*_OXA-10_	8	7.7
	*bla*_VEB-1_ + *bla*_OXA-10_	6	5.8
	*bla*_PSE_ + *bla*_OXA-10_	1	1
	*bla*_PER_ + *bla*_VEB-1_ + *bla*_OXA-10_	1	1
MBL^b^ encoding genes only (3)	*bla*_VIM_	3	2.9
ESBL + MBL encoding genes (4)	*bla*_OXA-10_ + *bla*_VIM_	1	1
	*bla*_OXA-10_ + *bla*_AIM_	2	1.9
	*bla*_PER_ + *bla*_OXA-10_ + *bla*_AIM_	1	1

^a^ESBL: Extended-spectrum β-lactamase

^b^MBL: Metallo-β-lactamase

More than half of the ESBL producers were isolated from pus (52.9%) while the rest were obtained from various sources: bronchial lavage (22.4%), urine (14.1%), sputum (9.4%), and blood (1.2%). Two of the three isolates possessing only MBL encoding genes were pus isolates and the third was obtained from bronchial lavage. All isolates harboring ESBL + MBL genotype combinations were pus isolates. Non-ESBL/non-MBL producers were mainly isolated from pus and bronchial lavage (41.7% each), followed by urine and blood culture (8.3% each) (Additional file 1: Table S3).

**Fig. 2:**
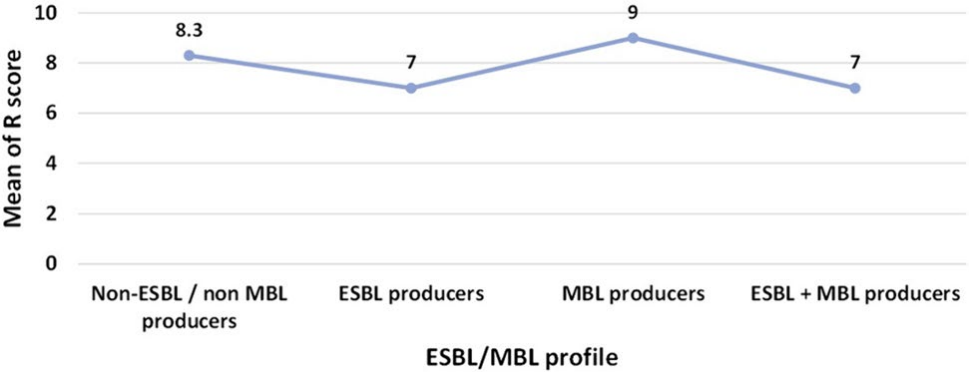
Distribution of resistance score (R score) mean values among *P. aeruginosa* ESBL/MBL profiles

All non-ESBL/non-MBL producing isolates were resistant to the tested carbapenems and quinolones and susceptible to colistin. Piperacillin-tazobactam, cefepime, and gentamicin showed similar activity against non-ESBL/non-MBL producers (8.3% susceptibility for each). The least active antibiotic against ESBL producers was imipenem followed by ciprofloxacin (11.8 and 16.5% susceptibility, respectively) while the most active agent was colistin followed by piperacillin-tazobactam (92.9 and 31.8% susceptibility, respectively). MBL producers exhibited 100% resistance rate to all of the tested antibiotics, except for colistin. Also, all the ESBL + MBL producers were susceptible to colistin, however, they were resistant to cefepime and gentamicin. Among the tested isolates, a non-significant association (*p*-value > 0.05) has been detected between the antimicrobial susceptibility and the ESBL/MBL profile (Table 3 & Additional file 1: Table S3). MBL producers displayed the highest R score mean value followed by the non-ESBL/non-MBL producers (R score mean values = 9 and 8.3, respectively). The same R score mean value of 7 was recorded for both ESBL producers and ESBL + MBL producers **(**Fig. 2**).**

**Table 3:** Antimicrobial susceptibility among *P. aeruginosa* isolates stratified by their ESBL/MBL profile

Antimicrobial agent	Susceptibility percentage^*a*^ (%) of isolates												*P*-value^d^
	Non-ESBL^b^/ non MBL producers (n = 12)			ESBL producers (n = 85)			MBL^c^ producers (n = 3)			ESBL + MBL producers (n = 4)			*P*-value
	S	I	R	S	I	R	S	I	R	S	I	R	
Piperacillin-tazobactam	8.3	25	66.7	31.8	36.5	31.8	0	0	100	25	25	50	0.086
Ceftazidime	16.7	25	58.3	27.1	5.9	67.1	0	0	100	25	0	75	0.281
Cefepime	8.3	0	91.7	17.6	2.4	80	0	0	100	0	0	100	0.853
Imipenem	0	0	100	11.8	3.5	84.7	0	0	100	25	0	75	0.735
Meropenem	0	0	100	21.2	5.9	72.9	0	0	100	25	25	50	0.22
Gentamicin	8.3	0	91.7	18.8	1.2	80	0	0	100	0	0	100	0.857
Ciprofloxacin	0	0	100	16.5	0	83.5	0	0	100	25	0	75	0.365
Levofloxacin	0	0	100	17.6	0	82.4	0	0	100	25	0	75	0.341
Moxifloxacin	0	0	100	18.8	0	81.2	0	0	100	25	0	75	0.317
Colistin	100	0	0	92.9	0	7.1	100	0	0	100	0	0	0.7

^a^S: sensitive, I: intermediate, and R: resistant

^b^ESBL: Extended-spectrum β-lactamase

^c^MBL: Metallo-β-lactamase

^d^The *p*-values indicate significance where *p < 0.05 (significant)

### Plasmid isolation

**Fig. 3:**
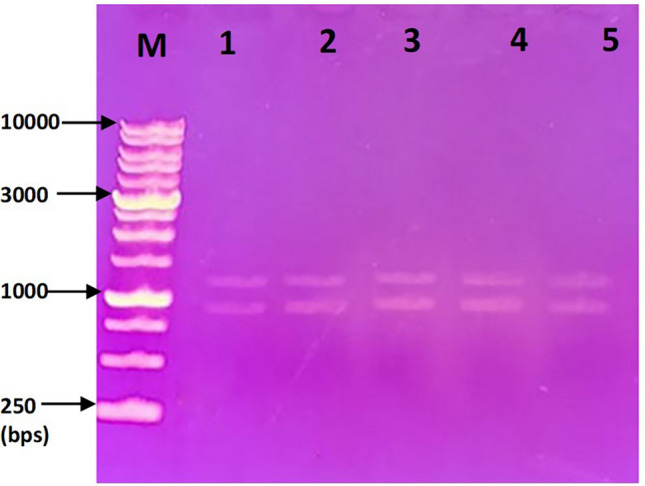
Plasmid profile of selected *P. aeruginosa* clinical isolates. M: 1 kb DNA ladder, lane 1: P23, lane 2: P100, lane 3: P101, lane 4: P108, and lane 5: P121

**Fig. 4:**
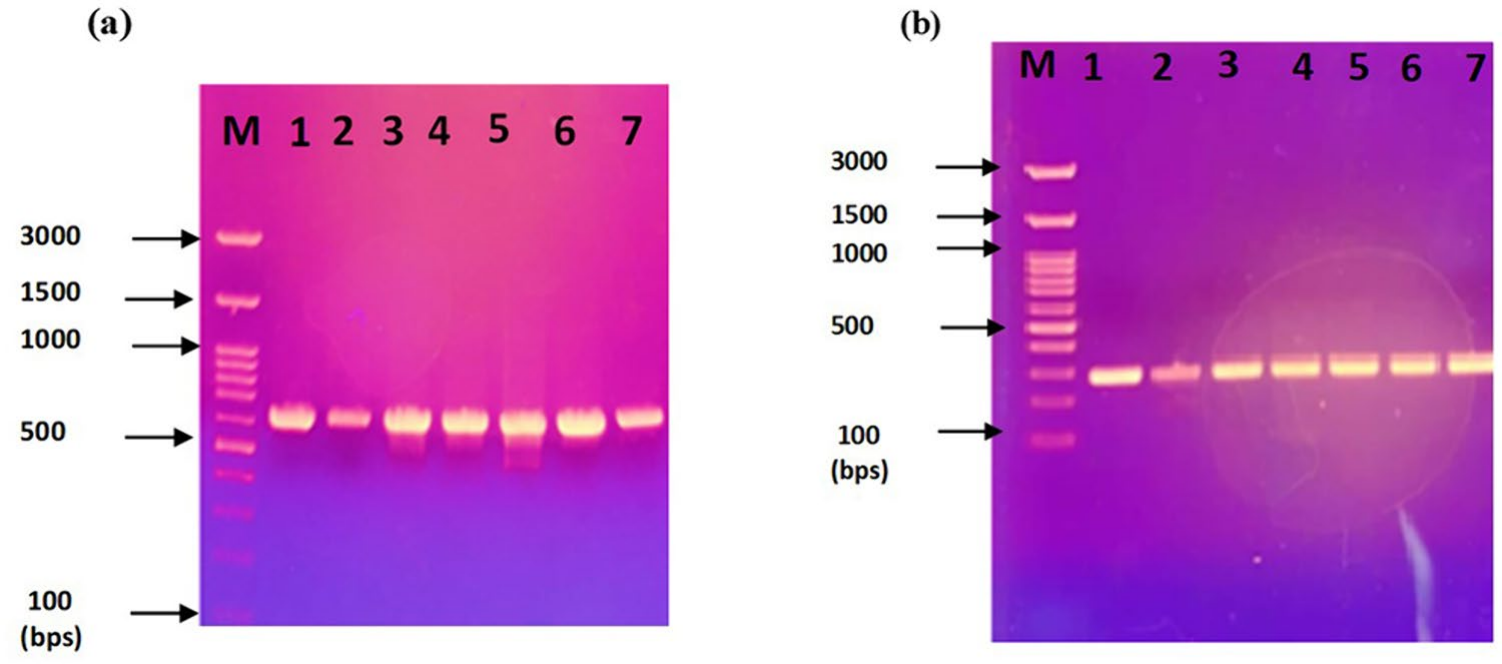
PCR products of **a** *bla*_VEB-1_ gene and **b** *bla*_OXA-10_ gene using plasmid preparations as templates. **a** *bla*_VEB-1_ gene (643 bps); M: a 100 bps DNA ladder, lane 1: P23, lane 2: P78, lane 3: P100, lane 4: P101, lane 5: P108, lane 6: P121, lane 7: P123, **b** *bla*_OXA-10_ gene (276 bps); M: a 100 bps DNA ladder, lane 1: P23, lane 2: P78, lane 3: P100, lane 4: P101, lane 5: P108, lane 6: P121, and lane 7: P123

Plasmids were isolated from ceftazidime-resistant *P*. *aeruginosa* isolates (P23, P78, P100, P101, P108, P121, and P123) that co-harbored both *bla*_VEB-1_ and *bla*_OXA-10_. After gel electrophoresis, two plasmid bands were detected among each of five (out of seven tested) plasmid preparations belonging to isolates P23, P100, P101, P108, and P121 (Fig. 3). However, plasmid preparations of P78 and P123 could not be visualized by gel electrophoresis. Positive PCR amplification of *bla*_VEB-1_ and *bla*_OXA-10_ from the seven plasmid preparations confirmed that both genes were plasmid-borne (Fig. 4a, b). The amplified PCR products of *bla*_VEB-1_ were sequenced and showed 100% similarity to the publically available sequence of VEB-1a (GenBank accession no. HM370393.1).

### Curing of the plasmids from the isolates carrying *bla*_VEB-1_ and *bla*_OXA-10_

Curing of the plasmids from the seven selected *P. aeruginosa* isolates was attempted using SDS and EtBr as curing agents. After selection on LB agar plates containing 0.5 × MIC of marker antimicrobial agents (ceftazidime, imipenem, gentamicin, and levofloxacin), all the colonies selected after treatment showed resistance to the tested antibiotics.

### Transformation of *bla*_VEB-1_ and *bla*_OXA-10_—carrying plasmids into chemically competent *E. coli*

The transformation of 5 plasmid preparations from P23, P100, P101, P108, and P121, visualized by gel electrophoresis, into chemically competent *E. coli* DH5α was successful. Plasmid preparation of isolate P101 showed the highest transformation efficiency (3.7 × 10^4^ CFU/μg plasmid DNA), whilst the lowest transformation efficiency was observed in case of plasmid preparation of isolate P108 (6.8 × 10^3^ CFU/μg plasmid DNA). All the selected tested transformants were ceftazidime-resistant showing more than 512-fold increase in MIC values except for transformants obtained from plasmid preparation of isolate P108 that showed more than 64-fold increase compared to the wild *E. coli* DH5α (Table 4). Furthermore, plasmids were isolated from selected transformants and were used as templates for PCR detection of both *bla*_VEB-1_ and *bla*_OXA-10_. Only *bla*_OXA-10_ was detected among the five plasmid preparations that were successfully transformed into *E. coli* DH5α (Additional file 2: Fig. S1).

**Table 4:** Transformation of plasmid preparations from selected *P. aeruginosa* isolates into *E. coli* DH5α competent cells

Plasmid preparation	Concentration (ng/μl) of the plasmid extract	No. of obtained transformants	Transformation efficiency (CFU^a^/μg DNA plasmid)	Susceptibility to ceftazidime (MIC^b^ in μg/ml) (S or R^c^)	
				Before transformation	After transformation
P23	63.2	267	1.6 X 10^4^	< 0.5 (S)	256 (R)
P100	32.9	138	1.6 X 10^4^	< 0.5 (S)	>256 (R)
P101	12.3	120	3.7 X10^4^	< 0.5 (S)	>256 (R)
P108	86.2	155	6.8 X 10^3^	< 0.5 (S)	32 (R)
P121	27	250	3.5 X 10^4^	< 0.5 (S)	>256 (R)

^a^CFU: colony forming unit

^b^MIC: minimum inhibitory concentration

^c^S: sensitive and R: resistant

## Discussion

Owing to the extensive use of antimicrobial agents and the continuous rise in the number of immunosuppressed patients, *P. aeruginosa* has become a leading cause of life-threatening Gram-negative hospital-acquired infections [38]. Among the most worrisome features of this pathogen is its remarkable ability to inherently resist various classes of antibiotics and acquire resistance to nearly all effective antimicrobial agents [39]. Consequently, this hampers the available treatment options, increases the health care costs, and elevates the burden of morbidity and mortality [40].

Resistance to each of cefepime, gentamicin, moxifloxacin, levofloxacin, ciprofloxacin, and imipenem was greater than 80% among the tested isolates. Higher resistance rates to cefepime (98.2%) and ceftazidime (91.2%) among *P. aeruginosa* isolates were formerly reported in Egypt [41]. In contrast, Farhan et al. [42] and Diab et al. [43] reported lower rates of resistance in Egypt to cefepime and ceftazidime, respectively. MIC values comparable to those obtained in the current study for both cephalosporins have been previously recorded in Egypt and Saudi Arabia [44, 45]. The 38.5% resistance rate to piperacillin-tazobactam reported here is lower than that documented by Diab et al*.* [43]. Yet, the MIC values of piperacillin-tazobactam among the tested isolates are close to those described by Basha et al. [44] and Ochoa et al. [46].

Despite the fact that carbapenems are among the most effective treatment choices against *P. aeruginosa* [5], imipenem was the least effective antibiotic in this study. This result is in line with that reported by Khorvash et al. [47], in Iran, who found imipenem resistance of 97.9% among the tested *P. aeruginosa* isolates. In Egypt, 78.3 and 72% resistance to imipenem were previously reported by Abaza et al. [40] and Diab et al. [43], respectively. On the contrary, our finding disagrees with Farhan et al. [42] and Elmaraghy et al. [48], from Egypt, who found that imipenem showed resistance rates of only 8 and 14.9%, respectively. In our study, meropenem also exhibited an elevated resistance rate reaching 76%. Similar to imipenem, high prevalence of meropenem resistance was formerly observed in Egypt [40, 43]. The obtained ranges of MIC values of imipenem and meropenem are consistent, to some extent, with those reported by Basha et al. [44].

Although aminoglycosides have been recognized as a significant treatment option for *P. aeruginosa* infections, the development of aminoglycoside resistance has been widely reported [49]. The current study showed that the resistance rate to gentamicin was 82.7%. This result is higher than the previously reported rates globally [50–52] (ranging between 16.8 and 28.5%) and in Egypt [42, 48, 53] (ranging from 6 to 44.8%). In the current study, gentamicin possessed the highest MIC_50_ and MIC_90_ values among all tested antibiotics. A comparable finding was reported by Basha et al. [44].

In this study, elevated resistance rates to the three tested fluoroquinolones: ciprofloxacin, levofloxacin, and moxifloxacin (85.6, 84.6 and 83.7%, respectively) were found. Similarly, in Egypt, Abaza et al. [40] reported 76.6% resistance rate to ciprofloxacin and Basha et al. [44] recorded 67% resistance level to levofloxacin. On the contrary, Abbas et al*.* [53] reported lower resistance rates to ciprofloxacin and levofloxacin (8 and 6%, respectively). Also, in contrast with our findings, a Chinese study recorded that more than 60% of the *P. aeruginosa* isolates were susceptible to the same three fluoroquinolones tested here [54]. Such discrepancies in resistance levels might be due to the difference in antibiotic consumption rates and selective pressure in distinct geographical regions.

Colistin was the most effective antimicrobial agent against the present collection of isolates recording a susceptibility rate of 94.2%, and the lowest MIC_50_ and MIC_90_ values (< 0.5 and 2 µg/ml, respectively). Similarly, in an Iranian study conducted by Malekzadegan et al. [55], the tested *P. aeruginosa* isolates showed susceptibility to colistin with MIC_50_ and MIC_90_ of 0.5 and 1 µg/mL, respectively.

The pairwise correlation between the susceptibility patterns of the tested clinical isolates to the antimicrobial agents under investigation was studied. Highly significant correlations have been noticed between all of the tested pairs of β-lactam antibiotics (r_s_ ≥ 0.523; *p*-value < 0.001) as well as the studied pairs of fluoroquinolones (r_s_ ≥ 0.735; *p*-value < 0.001). Such highly significant, but partial, correlations could be attributed to the potential common resistance mechanisms to these antimicrobial agents belonging to the same class, while highlighting strain variation, differences in antibiotic chemical structures and enzyme specificities [22]. In addition, highly significant correlations (*p*-value < 0.001), with r_s_ ranging between 0.84 and 0.64, have been detected between the three tested members of fluoroquinolones and both carbapenems. Such fluoroquinolone-carbapenem significant correlations could be explained according to the fact that, in *P. aeruginosa*, exposure to a fluoroquinolone stimulates carbapenem resistance owing to transcriptional downregulation of the porin OprD as well as efflux pumps upregulation [56]. On the contrary, the correlations between colistin and all the other tested antibiotics were non-significant with r_s_ ≤ 0.124. This could be due to the different underlying mechanisms by which bacteria acquire resistance to colistin relative to other classes of antibiotics.

Despite the high level of resistance to ceftazidime and cefepime, none of the tested isolates showed positive results for ESBL production when phenotypically screened using conventional DDST and PCDDT. Similarly, Mansour et al. [57] demonstrated that none of the tested *P. aeruginosa* isolates were ESBL producers by the conventional DDST test. On the contrary, a noticeable level of ESBL detection (36%), by phenotypic DDST, was reported by Farhan et al*.* [42]. Generally, the available methods for the phenotypic detection of ESBLs are unreliable. This issue has been previously discussed by Zafer et al*.* [58], Jiang et al*.* [59], and Poulou et al*.* [60] who documented the uncertainty and unreliability of the current phenotypic methods for detection of ESBLs in *P. aeruginosa* and that these methods may give false-negative results. The presence of a combination of ESBL and *ampC* genes may lead to failure in the phenotypic detection of ESBLs by conventional DDST. Also, the false-negative results obtained from DDST despite the phenotypic resistance observed could be explained by the presence of other resistance mechanism, such as efflux and impermeability [24].

The phenotypic detection of class B MBLs (carbapenemases), using the combined imipenem-EDTA disc test, revealed that all the imipenem-resistant isolates were MBL producers. In Egypt, a similar finding was reported by Basha et al*.* [44] who found that all of the tested carbapenem resistant *P*. *aeruginosa* isolates, displaying the highest MIC values to imipenem and meropenem, showed positive results using this phenotypic test.

Molecular detection of selected β-lactamase encoding genes of different Ambler classes was performed using the PCR technique. The isolates were screened for class A β-lactamases; *bla*_PER_, *bla*_PSE_, and *bla*_VEB-1_. *bla*_PER_, an ESBL first reported in Turkey in 1991, showed a prevalence of 11.5% among the tested isolates; a finding comparable to that previously recorded by Gaballah et al*.* [61]. In contrast, Strateva et al. [32] noted the absence of *bla*_PER_ among Bulgarian *P. aeruginosa* isolates. *bla*_PSE,_ was detected only in one isolate. Also, Gaballah et al. [61] found that only 2 out of 30 *P. aeruginosa* isolates harbored *bla*_PSE_. On the other hand, high occurrence rate of *bla*_PSE_ was previously reported in Egypt [53] and Taiwan [62]. Among our isolates, 6.7% harbored *bla*_VEB-1_. Such low prevalence of *bla*_VEB-1_ is comparable to that reported in an Egyptian study conducted by Zafer et al. [58]. *bla*_VEB_ was also detected in Egypt by Gaballah et al. [61], yet at a prevalence rate of 33% among the tested *P. aeruginosa* isolates. On the other hand, in another Egyptian study, Abbas et al*.* [53] noticed the absence of *bla*_VEB_ among the tested *P. aeruginosa* isolates. Among numerous acquired β-lactamase enzymes, the *bla*_VEB-1_ possesses the most considerable clinical significance because it mediates resistance to oxyimino β -lactams [63]. Furthermore, *bla*_VEB-1_ is one of the most commonly detected ESBLs among *P. aeruginosa* from the Middle East including Saudi Arabia, Kuwait, and Iran [64].

Regarding class B MBLs, four genes were investigated among the *P. aeruginosa* isolates: *bla*_IMP_, *bla*_VIM_, *bla*_NDM_ and *bla*_AIM_. VIM β-lactamases are the most common type of MBLs in *P. aeruginosa* [65]. However, in the current study, *bla*_VIM_ was detected only among 3.8% of the tested isolates. In Egypt, a higher prevalence rate of *bla*_VIM_ was recorded by Farhan et al. [42]. In contrast, other Egyptian studies reported the absence of *bla*_VIM_ among *P. aeruginosa* isolates [40, 53]. *bla*_IMP_ was not detected at all. A similar finding has been reported in several studies globally [32, 45, 62, 66, 67] and in Egypt [43, 44, 61]. On the contrary, Abaza et al. [40] and Farhan et al. [42] reported 36.7 and 42.8% prevalence of *bla*_IMP_ among their tested *P. aeruginosa* isolates, respectively. None of the tested isolates in this study possessed *bla*_NDM_. The absence of that gene among *P. aeruginosa* strains has been previously recorded in Egypt [61] and Saudi Arabia [45]. Yet, Basha et al*.* [44] and Mukaya et al. [68] reported that *bla*_NDM_ was the most prevalent MBL (90.9 and 51.9%, respectively) among the tested *P. aeruginosa* isolates in their studies. To the best of the authors’ knowledge, this is the first study of the prevalence of *bla*_AIM_ in Egypt. Few reports are available worldwide about the occurrence of this newly identified MBL in *P. aeruginosa*. In the current investigation, only 3 isolates (2.9%) harbored this gene. Adelaide imipenemase 1 (AIM-1) was first reported, in 2008, in Adelaide, Australia. It has been suggested that *bla*_AIM-1_ gene, in *P. aeruginosa*, originated from *Pseudoxanthomonas mexicana*, a non- pathogenic environmental bacterium [69]. Although this gene has been associated with a high-level resistance phenotype as well as a wide prevalence among environmental, non-pathogenic bacteria, it is scarcely detected among pathogenic bacterial communities. *P. aeruginosa* harboring *bla*_AIM-1_ gene has been infrequently, though repeatedly, detected in certain region; Adelaide [69]. Nevertheless, in Iran, Neyestanaki et al*.* [31] reported 1% prevalence of *bla*_AIM_ among the tested *P. aeruginosa* isolates. In Iraq, a 37.5% prevalence rate of *bla*_AIM_ has been recorded among the tested isolates [70]. However, in another Iranian study, the complete absence of *bla*_AIM_ was recorded [47].

The results of the phenotypic detection of MBLs revealed that 87.5% of the tested isolates were MBL producers. Based on the PCR results, only 7 isolates carried MBL genes. This might suggest that the MBL enzymatic activity observed by phenotypic IMP-EDTA CDT might be due to other MBL genes not investigated in this study. Similarly, Abbas et al. [53] and Gaballah et al. [61] found that some IMP-EDTA CDT-positive isolates did not harbor the tested MBL gene(s). However, it is worthy to note that EDTA has been recognized as an effective permeabilizer against* P. aeruginosa* [71] and, owing to its metal chelating properties, EDTA can increase the susceptibility of *Acinetobacter* spp. to various antibiotics including imipenem [72]. This might result in false-positive results of IMP-EDTA CDT. Marra et al. [73] documented that EDTA may cause false-positive detection rate of MBLs reaching 69.6%. According to Chu et al. [74], the IMP-EDTA disc method should be applied with caution for the detection of MBLs where this test is suitable for preliminary screening. However, it should not be utilized as the only indicator for detecting MBLs.

*bla*_OXA-10_, a class D ESBL, displayed the highest occurrence rate (83.7%) among the tested isolates. *bla*_OXA-10_ is one of the most prevalent ESBL genes in *P. aeruginosa* in several countries, including Iran, Bulgaria, Korea, and Palestine [31, 32, 75, 76]. In Egypt, Zafer et al. [58] revealed that *bla*_OXA-10_ -like genes were the most common ESBLs among the tested *P. aeruginosa* isolates with a prevalence rate exceeding 40%.

It was noted that the molecular detection of ESBL genes was not consistent with the phenotypic screening where 85.6% of the isolates were shown molecularly to carry ESBL genes, yet none of the isolates were found to be ESBL producers phenotypically. In accordance, Aktaş et al. [77] mentioned that DDST might fail in the detection of isolates producing PER-1- and OXA-derived enzymes. Also, in their study, only 37% of the PER-1-positive strains were DDST-positive. Moreover, Zafer et al. [58] found that, upon phenotypic screening for ESBL production, only 9 isolates showed positive results while 20 and 5 isolates, respectively, were OXA-10-positive and VEB-positive by PCR. Similar differences between the phenotypic and genotypic prevalence of β-lactamases among *Acinetobacter baumannii* isolates was noted by our group [78]. Such findings confirm the unreliability of the phenotypic methods singly and/or alone for the detection of β-lactamases as they may lead to false-negative or false-positive results, thus affecting the choice of the proper antibiotic therapy and in turn resulting in treatment failure.

In this study, four ESBL-genotype combinations were detected among 16 (15.4%) of the tested isolates. The co-existence of more than one ESBL encoding gene among *P*. *aeruginosa* isolates has been previously reported [12, 79]. In addition, in the current investigation, ESBL + MBL- genotype combinations were detected among 4 isolates (3.8%). Similarly, the co-harboring of ESBL and MBL encoding genes among *P. aeruginosa* isolates have been recorded in Egypt [61], India [5], and Saudi Arabia [80]. The co-production of various classes of β-lactamases by a single clinical isolate may give rise to serious diagnostic challenges and result in therapeutic failure. Accordingly, expeditious recognition of β-lactamase production might help in the implementation of suitable antibiotic therapy as well as infection control strategies [81, 82].

In an Indian study conducted by Chaudhary and Payasi [5], the susceptibility to different antibiotics among *P. aeruginosa* isolates with various ESBL/MBL profiles was studied. In comparison to their study, lower susceptibility rates to imipenem and meropenem were detected among our ESBL producers while lower percentages of resistance to both carbapenems were observed among our ESBL + MBL producers. Also, our ESBL producers showed lower percentages of susceptibility to piperacillin-tazobactam. Compared to the findings of Chaudhary and Payasi [5], our ESBL producers and ESBL + MBL producers showed a higher resistance rate to cefepime. For ceftazidime, a higher resistance rate was detected among our ESBL producers while a lower resistance rate was noticed among the ESBL + MBL producers. Also, a lower percentage of resistance against piperacillin-tazobactam was detected among our ESBL + MBL producers. In line with our findings, Chaudhary and Payasi [5] reported that all MBL producers were resistant to piperacillin-tazobactam, ceftazidime, cefepime, imipenem, and meropenem. Against these five antibiotics, our non-ESBL/non-MBL producers showed resistance rates ranging between 58.3 and 100%. On the contrary, in their study, the non-ESBL producers showed 100% susceptibility to these antibiotics [5].

Upon comparing the resistance profiles of MBL producers and ESBL + MBL producers in our study, MBL producers showed higher resistance rates against piperacillin-tazobactam, ceftazidime, imipenem, meropenem, and the three tested fluoroquinolones. Similarly, Chaudhary and Payasi [5] reported higher percentages of resistance against piperacillin-tazobactam, ceftazidime, imipenem and meropenem among MBL producers compared to ESBL + MBL producers. Although a higher resistance rate against cefepime among MBL producers was recorded in their study [5], in ours, equal percentages of susceptibility towards cefepime, gentamicin and colistin were detected among both groups. In our study, higher resistance levels against piperacillin-tazobactam, cefepime, imipenem, meropenem, gentamicin, and the three tested fluoroquinolones were detected in case of non-ESBL/non MBL producers when compared to ESBL producers. On the contrary, Chaudhary and Payasi [5] recorded higher susceptibility rates (100%) to piperacillin-tazobactam, cefepime, imipenem and meropenem among non-ESBL producers compared to ESBL producers. In our study, lower percentages of resistance against both ceftazidime and colistin were noticed in case of non-ESBL/non MBL producers compared to ESBL producers. In comparison with ESBL producers, a higher susceptibility rate (100%) to ceftazidime among non-ESBL producers was reported by Chaudhary and Payasi [5].

Ceftazidime, a third-generation cephalosporin, has been commonly used for the treatment of *P. aeruginosa* infections. However, the rising rate of ceftazidime resistance has led to poor therapeutic outcomes [83]. The widespread of ceftazidime-resistant *P. aeruginosa* clinical isolates has been reported globally [45, 46, 83, 84] and in Egypt [41, 42, 44]. Mainly, ceftazidime resistance can be mediated by the production of various β-lactamases including ESBL, MBL and infrequently AmpC-β-lactamases [83]. High prevalence rate of *bla*_OXA-10_ and *bla*_VEB-1_ -like genes among ceftazidime-resistant *P. aeruginosa* clinical isolates has been previously reported [84, 85], in accordance with the present findings.

In this study, plasmid isolation from the seven ceftazidime-resistant isolates harboring *bla*_VEB-1_ and *bla*_OXA-10_ was done. After gel electrophoresis, two plasmid bands were detected in 5 out of 7 isolates. The PCR results revealed that all the plasmid preparations harbored both *bla*_VEB-1_ and *bla*_OXA-10_. This agrees with Maurya et al. [86] and Behbahani et al. [87] who documented that both *bla*_VEB_ and *bla*_OXA-10_ genes, respectively, are plasmid mediated. Furthermore, to gain more insight about the role of *P. aeruginosa* plasmids in mediating antibiotic resistance, plasmid curing and transformation experiments were carried out to confirm that the antibiotic resistance genes were plasmid mediated. In the current work, EtBr and SDS, at different tested concentrations, were used as curing agents. All seven isolates remained resistant to the tested antimicrobial agents. This could be attributed to the high copy number of these plasmids [36], or the contribution of chromosomal mediated resistance mechanisms among the tested isolates. In contrast, Thomas et al. [36] reported successful attempts of curing with 10% SDS and loss of resistance to ceftazidime, imipenem, and gentamicin among all the tested strains. However, using EtBr as curing agent, ceftazidime resistance was only lost at a concentration of 200 µg/ml among 68.7% of the tested strains.

The plasmid preparations from the seven isolates harboring *bla*_VEB-1_ and *bla*_OXA-10_ were transformed into chemically competent *E. coli* DH5α. The transformation of only five plasmid preparations was successful with high transformation efficiencies. The selected tested transformants showed high resistance to ceftazidime and harbored plasmids carrying *bla*_OXA-10_. In a previous investigation, Maurya et al*.* [88] reported the successful transformation of *E. coli* JM107 cells with a plasmid harboring *bla*_OXA-10_. The high level of acquired resistance to ceftazidime in the tested transformants, in this investigation, may be due to the uptake of plasmid encoding *bla*_OXA-10_ and/or the co-presence of other ESBLs genes not explored in this study. The failure of the detection of *bla*_VEB-1_ in the obtained transformants of *E. coli* may be due to the inability of the plasmids carrying the gene to replicate into the recipient cells, or due to the large size of the plasmid carrying the gene leading to unsuccessful transfer of that plasmid. The impact of increasing plasmid size on the decline of the transformation efficiency has been previously reported [89, 90].

## Conclusions

The current study shows that resistance levels to carbapenems, cephalosporins, and fluoroquinolones among *P. aeruginosa* clinical isolates are alarming, thus rendering these infections hard to treat. This necessitates the implementation of proper antibiotic usage policies to prevent the injudicious use of antimicrobial agents in hospitals. Moreover, ESBL-genotype combinations as well as ESBL + MBL- genotype combinations have been detected among the tested isolates, thus leading to poor therapeutic outcomes. The expeditious characterization of ESBLs and MBLs is mandatory to hinder their dissemination, impede the spread of multidrug resistant pathogens, and reduce the associated mortality rates among affected patients.

## Supplementary Information

Below is the link to the electronic supplementary material.

Supplementary file1 (DOCX 18 KB)

Supplementary file2 (DOCX 16 KB)

Supplementary file3 (XLSX 16 KB)

Supplementary file4 (TIF 474 KB)

## Data Availability

Data generated or analysed during this study are included in this published article and its supplementary information files.

## Abbreviations

ESBLs: Extended-spectrum β-lactamases
MBLs: Metallo-β-lactamases
PCR: Polymerase chain reaction
AMUH: Alexandria Main University Hospital
TSI: Triple sugar iron
MIC: Minimum inhibitory concentration
CLSI: Clinical and Laboratory Standards Institute
R score: Resistance score
DDST: Double disc synergy test
PCDDT: Phenotypic confirmatory disc diffusion test
IPM-EDTA CDT: Combined imipenem-EDTA disc test
DNA: Deoxyribonucleic acid
TAE: Tris Acetate EDTA
EDTA: Ethylenediamine tetraacetic acid
UV: Ultraviolet
LB: Luria–Bertani
EtBr: Ethidium bromide
SDS: Sodium dodecyl sulfate
BLAST: Basic Local Alignment Search Tool
CFU: Colony forming unit
