# Supplementary material for: Phenotypic and molecular characterization of extended spectrum- and metallo- beta lactamase producing Pseudomonas aeruginosa clinical isolates from Egypt
Source: Infection. 2024 Jun 2;52(6):2399–414. doi: 10.1007/s15010-024-02297-8 (PMC11621155; doi:10.1007/s15010-024-02297-8)
Supplement: Supplementary file 1 — Supplementary file1 (DOCX 18 KB) [file 15010_2024_2297_MOESM1_ESM.docx]

**Table S1: Oligonucleotide primers sequence and amplicon size.**

| Target gene | Primer name | Nucleotide sequence (5′→3′) | Size of the  amplicons (bps) |
| --- | --- | --- | --- |
| *bla*_PER_ [29] | PER-F | ATGAATGTCATTATAAAAGC | 920 |
|  | PER-R | AATTTGGGCTTAGGGCAGAA |  |
| *bla*_VIM_ [30] | VIM-F | ATGTTAAAAGTTATTAGTAGT | 801 |
|  | VIM-R | CTACTCGGCGACTGAGCGAT |  |
| *bla*_IMP_  [31] | IMP-F | GGAATAGAGTGGCTTAAYTCTC | 232 |
|  | IMP-R | GGTTTAAYAAAACAACCACC |  |
| *bla*_OXA-10_ [31] | OXA-10-F | TCAACAAATCGCCAGAGAAG | 276 |
|  | OXA-10-R | TCCCACACCAGAAAAACCAG |  |
| *bla*_AIM_ [31] | AIM-F | CTGAAGGTGTACGGAAACAC | 322 |
|  | AIM-R | GTTCGGCCACCTCGAATTG |  |
| *bla*_NDM_ [31] | NDM-F | GGTTTGGCGATCTGGTTTTC | 621 |
|  | NDM-R | CGGAATGGCTCATCACGATC |  |
| *bla*_PSE_  [31] | PSE-F | AATGGCAATCAGCGCTTC | 698 |
|  | PSE-R | GCGCGACTGTGATGTATA |  |
| *bla*_VEB-1_ [32] | VEB-1-F | CGACTTCCATTTCCCGATGC | 643 |
|  | VEB-1-R | GGACTCTGCAACAAATACGC |  |
