# Supplementary material for: Phenotypic and molecular characterization of extended spectrum- and metallo- beta lactamase producing Pseudomonas aeruginosa clinical isolates from Egypt
Source: Infection. 2024 Jun 2;52(6):2399–414. doi: 10.1007/s15010-024-02297-8 (PMC11621155; doi:10.1007/s15010-024-02297-8)
Supplement: Supplementary file 2 — Supplementary file2 (DOCX 16 KB) [file 15010_2024_2297_MOESM2_ESM.docx]

**Table S2: PCR amplification conditions for genes encoding selected β-lactamases.**

| β-lactamases class | Target gene | PCR thermal cycling conditions | | | | |
| --- | --- | --- | --- | --- | --- | --- |
|  |  | **Initial denaturation** | **30 cycles** | | | **Final extension** |
|  |  |  | **Denaturation** | **Annealing** | **Extension** |  |
| Class A ESBL *^a^* genes | *bla*_PER_ | 94 °C/5 min | 94 °C/40 sec | 47 °C/1 min | 72 °C/2 min | 72 °C/10 min |
|  | *bla*_PSE_ |  |  | 48 °C/1 min |  |  |
|  | *bla*_VEB-1_ |  |  | 54 °C/1 min |  |  |
| Class B MBL *^b^* genes | *bla*_IMP_ | 94 °C/5 min | 94 °C/40 sec | 55 °C/1 min | 72 °C/2 min | 72 °C/10 min |
|  | *bla*_VIM_ |  |  | 53 °C/1 min |  |  |
|  | *bla*_AIM_ |  |  | 50 °C/1 min |  |  |
|  | *bla*_NDM_ |  |  | 50 °C/1 min |  |  |
| Class D ESBL genes | *bla*_OXA-10_ | 94 °C/5 min | 94 °C/40 sec | 55 °C/1 min | 72 °C/2 min | 72 °C/10 min |

*^a^* ESBL: Extended-spectrum β-lactamases.

*^b^* MBL: Metallo-β-lactamases.
