# Supplementary figures and images for: Phenotypic and molecular characterization of extended spectrum- and metallo- beta lactamase producing Pseudomonas aeruginosa clinical isolates from Egypt
Source: Infection. 2024 Jun 2;52(6):2399–414. doi: 10.1007/s15010-024-02297-8 (PMC11621155; doi:10.1007/s15010-024-02297-8)

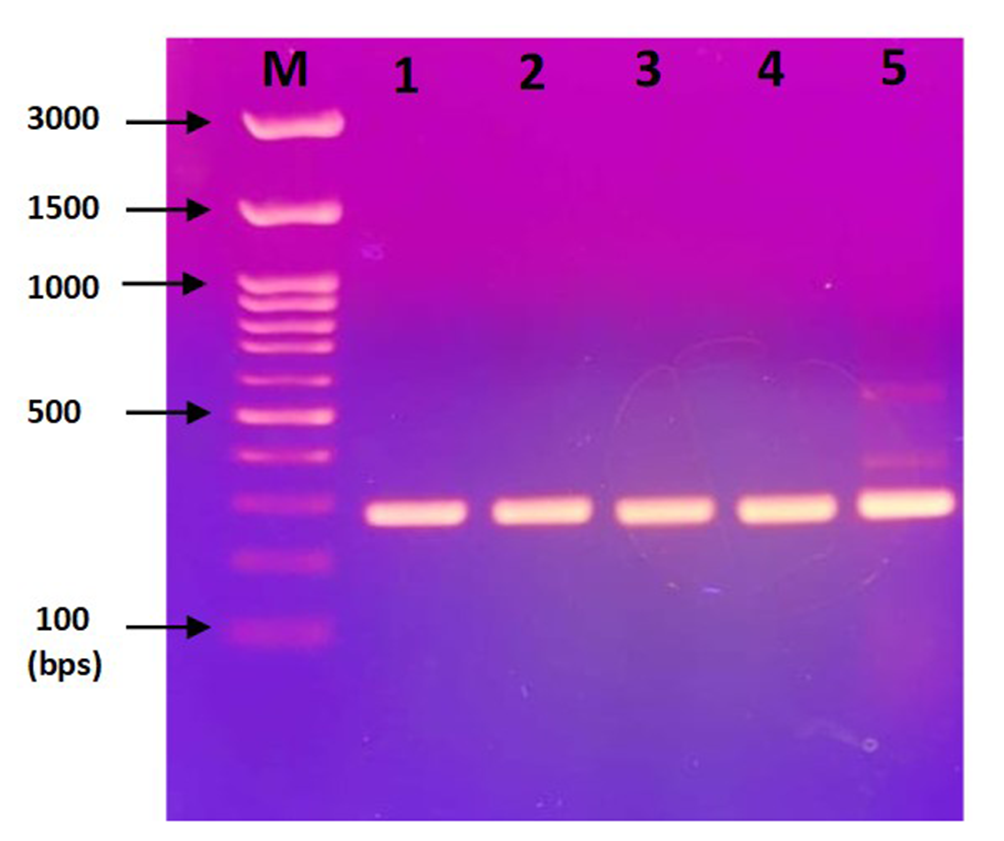

Supplement: Supplementary file 4 — Supplementary file4 (TIF 474 KB) [file 15010_2024_2297_MOESM4_ESM.tif]
